# Increased prevalence of methicillin-resistant *Staphylococcus aureus *nasal colonization in household contacts of children with community acquired disease

**DOI:** 10.1186/1471-2334-12-45

**Published:** 2012-02-20

**Authors:** Yaseen Rafee, Nahed Abdel-Haq, Basim Asmar, Tanaz Salimnia, Celine Vidaillac Pharm, Michael J Rybak Pharm, Muhammad Amjad

**Affiliations:** 1 Division of Infectious Diseases, Children's Hospital of Michigan, Detroit, MI, USA; 2 Carman and Ann Adams Department of Pediatrics, Wayne State University, Detroit, MI, USA; 3 Wayne State University, Detroit, MI, USA; 4 Anti-Infective Research Laboratory, Eugene Applebaum College of Pharmacy and Health Sciences, Detroit, MI, USA; 5 School of Medicine, Wayne State University, Detroit, MI, USA; 6 Department of Clinical Laboratory Sciences, Marshall University, Huntington, WV, USA

**Keywords:** MRSA, Children, Nasal colonization

## Abstract

**Background:**

To measure Methicillin-resistant *Staphylococcus aureus *(MRSA) nasal colonization prevalence in household contacts of children with current community associated (CA)-MRSA infections (study group) in comparison with a group of household contacts of children without suspected *Staphylococcus aureus *infection (a control group).

**Methods:**

This is a cross sectional study. Cultures of the anterior nares were taken. Relatedness of isolated strains was tested using pulse field gel electrophoresis (PFGE).

**Results:**

The prevalence of MRSA colonization in the study group was significantly higher than in the control group (18/77 (23%) vs 3/77 (3.9%); p ≤ 0.001). The prevalence of SA colonization was 28/77 (36%) in the study group and 16/77 (21%) in the control group (p = 0.032). The prevalence of SA nasal colonization among patients was 6/24 (25%); one with methicillin-susceptible *S. aureus *(MSSA) and 5 with MRSA. In the study (patient) group, 14/24 (58%) families had at least one household member who was colonized with MRSA compared to 2/29 (6.9%) in the control group (p = 0.001). Of 69 total isolates tested by PFGE, 40 (58%) were related to USA300. Panton-Valetine leukocidin (PVL) genes were detected in 30/52 (58%) tested isolates. Among the families with ≥1 contact colonized with MRSA, similar PFGE profiles were found between the index patient and a contact in 10/14 families.

**Conclusions:**

Prevalence of asymptomatic nasal carriage of MRSA is higher among household contacts of patients with CA-MRSA disease than control group. Decolonizing such carriers may help prevent recurrent CA-MRSA infections.

## Background

Community-associated methicillin-resistant *Staphylococcus aureus *(CA-MRSA) infections are commonly recognized in persons without traditional risk factors, such as dialysis, intravenous permanent catheters, or intravenous drug abuse [1-5]. CA-MRSA is known to cause predominantly skin and soft-tissue infections, but can also cause other severe community associated infections like myositis, pyomyositis, osteomyelitis and bacteremia [6-10]. Furthermore, CA-MRSA infections tend to be recurrent. In one study, a recurrence rate of 15% was noted among adults [11]; whereas children had recurrent infections of 12-28% based on data from two separate reports [12,13].

Studies have shown that colonization with MRSA often precedes infection [14,15]. Ellis et al. reported that 38% of participants, who were initially colonized with CA-MRSA developed skin and soft tissue infection within the 8-10 weeks study period [16]. Furthermore, the prevalence of colonization with CA-MRSA appears to be increasing in parallel to increasing infections [6].

Despite limited data on intrafamilial transmission and nasal colonization of family members of patients with CA-MRSA infections [17-21], some experts recommend identifying possible household carriers of *S. aureus *in order to decolonize them by using mupirocin nasal ointment [22]. Whether routine decolonization of all family contacts of patients with recurrent CA-MRSA skin infections is needed remains unclear. Studies have shown that family members can serve as reservoirs for MRSA and that transmission can occur between family members including young children [18,19,21]. However, the prevalence of colonization of family contacts of patients with active CA-MRSA infections is unclear and no controlled studies that included all contacts within families have been previously reported. In addition, molecular typing data from these familial MRSA transmissions is limited [19,21]. Assessing the prevalence of colonization with CA-MRSA among family members of patients with CA-MRSA infections is needed in order to evaluate and implement prevention strategies. The purpose of our study was to investigate the prevalence of MRSA nasal colonization in household contacts of patients with CA-MRSA infections in comparison with household contacts of unaffected individuals in our community at large. In addition, we studied the relatedness of isolated strains using pulse field gel electrophoresis (PFGE).

## Methods

### Study design

This was a cross sectional study performed at Children's Hospital of Michigan (Detroit, MI). The study was approved by the Institutional Review Board at Wayne State University. Informed consent was obtained from all participants/legal guardians. The study group consisted of all household contacts of patients aged less than 21 years with CA-MRSA infections who were admitted to our hospital during the period October 1, 2007 to October 30, 2008. The control group consisted of patients without suspected staphylococcal infections admitted into our hospital and all their household contacts during the same time period. CA-MRSA infection was defined as infection caused by MRSA isolate cultured within 48 hours of admission. Participants in the study group were identified through prospective monitoring of the daily admission census and the culture results of the microbiology laboratory. For each patient with CA-MRSA infection enrolled, another patient of similar age without a suspected staphylococcal infection and not receiving antibiotics was identified and enrolled with all household contacts.

### Exclusion criteria

In both study and control groups, families were excluded if a household contact had received, during the preceding 8 weeks prior to participation, either intranasal antibiotic ointment including mupirocin, or antistaphylococcal antibiotics including clindamycin, cephalexin, cefazolin, oxacillin, dicloxacillin, trimethoprim/sulfamethoxazole, linezolid or vancomycin. Furthermore, in the control group, we excluded patients who have suspected active *S. aureus *infection and those who have risk factors for health-care associated MRSA infection. These include patients with chronic medical problems such as diabetes, kidney failure, or malignancy, and patients with frequent hospitalizations. Patients who have a family member known to have history of recurrent (≥2 episodes) or active skin or soft tissue infections were excluded from the control group.

### Data collection

After obtaining the appropriate consent, guardians of each study subject were interviewed by a study investigator. Data collected, included age, gender, ethnicity, the primary job of the working household member, any history of recent hospitalization, surgery, dialysis, a permanent indwelling catheter or percutaneous medical device, a positive culture for MRSA prior to this infection, history of recurrent soft tissue infection, antibiotic use, residence in a long -term care facility within the previous 12 months for patients and household contacts, sport or gym participation and volunteer time at a health related facility or day care center. Antibiotic susceptibility results of the all CA-MRSA isolates were also recorded. Based on recent published data [21], we hypothesized that the prevalence of CA-MRSA nasal colonization will be 20% in the household contacts of the CA-MRSA infected patients and 5% in the control group. Based upon this proportional effect size difference a sample size of 76 subjects in each study group would provide power of 80.5% with alpha set at 0.05, two-tailed. Sample Power Version 2.0 was used to calculate the sample size.

### Statistics

A non-parametric Fisher's Exact test was used to examine the difference in colonization rates between household members of patients with CA-MRSA infection and those in the control group. All statistical procedures were conducted using SPSS Version 15.0. To examine possible predictor variables of patients with CA-MRSA infections, as opposed to those patients who do not have the infection, a series of univariate and multivariate tests were conducted.

### Laboratory investigation

Using moistened double cotton swabs [(BBL CultureSwab Collection and Transport System; Becton, Dickinson and Company, Spark, MD, USA)], cultures were obtained from both anterior nares of each participant. For children with CA-MRSA infections, nasal swabs were obtained soon after identification of culture results from primary site of infection; all had received at least 48 hrs of antibiotic therapy prior to obtaining cultures. *Staphylococcus aureus *strain identification and antibiotic susceptibility testing were performed using standard laboratory procedures, including colonial morphology, gram stain, catalase test, tube coagulase test of citrated rabbit plasma, and the biochemical reactions in the Microscan Walk-Away (W. Sacramento CA, USA). DNA was extracted from MRSA (infective and nasal) isolates using UltraClean Microbial DNA isolation kit (Mo Bio laboratories, Solana Beach, Calif.). The multiplex PCR for *mec *element type assignment was performed according to the protocol developed by Oliveira et al. [23]. Detection of PVL genes, *LukS-PV *and *LukF-PV *was performed by PCR using primers described by Lina et al. [24]. *S. aureus *isolates were evaluated in Pulse Field Gel Electrophoresis (PFGE) using *Sma*I-digested DNA, as described previously [25]. Gels were run at 6 V/cm, 14°C, at an included angle of 120°, on a 1.2% agarose gel with pulse times of 5-35 sec for 21 hrs and strain relatedness was determined by visual inspection of the gel using the criteria of Tenover et al. [26], and dice's coefficient using BioNumerics Software (Version 4.6, Applied Maths, Saint-Martens-Latem, Belgium).

## Results

**Figure 1:**
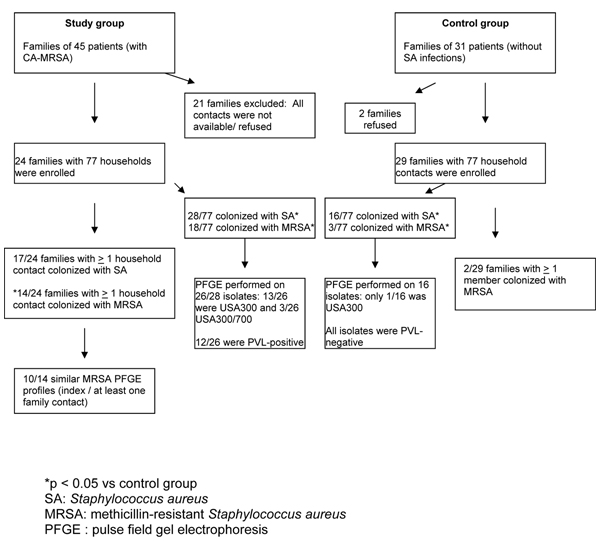
**Staphylococcal nasal colonization of household contacts in study and control groups**.

During the study period, families of 45 children younger than 21 years with CA-MRSA infection were approached. Only 24 families with a total of 77 household members available for screening were enrolled in the study group. The remaining families were excluded either because not all household members were available, or they refused to participate. Of the 24 patients with CA-MRSA infections, 15 had skin and soft tissue infection (SSTI), 4 had bone and joint infections, 3 had cervical lymphadenitis/abscess, one had chronic suppurative otitis media (CSOM) and one had spinal epidural abscess. Two of these patients were bacteremic, one with osteomyelitis and the other one with SSTI. In the control group, 31 families were approached. Only 29 families with a total of 77 household contacts were enrolled. Two families refused to participate (Figure 1). The mean size of the family members was 3.3 with standard deviation (SD) of 2.13 (Range 2-12) and 2.6 with SD of 1.02 (Range 2-6) in the study group and the control group, respectively (p > 0.5).

Demographics and the characteristics of household contacts in both groups are shown in Table 1. The mean age of patients in the study group was 4.47 yrs compared to 5.67 yrs in the control group (p = 0.656). The mean age of household contacts was 22.64 yrs in the study group compared to 26.20 yrs in the control group (p = 0.124). There were more African Americans in the control group than the study group (p = 0.001). Household contacts in both groups were similar with regard to history of eczema, history of antibiotic use in the preceding 6 months, or contact with a health care worker. Household contacts in the study group were more likely to report a history of sharing towels or having contact with someone with a history of IV drug abuse, incarceration, or homelessness (Table 1).

**Table 1:** Demographics and findings in the study and control groups

Characteristic	Contact gp (N77)	Control gp (N77)	*P*-value
Mean age (yrs)	22.64	26.20	0.124

Male (%)	45.2	54.8	0.33

African American (%)	38.7	61.3	0.001

Eczema (%)	2 (2.6)	4(5.2)	0.67

Day care attendance (%)	1(1.3)	6(7.8)	0.058

Sharing towels (%)	35(45.5)	16(20.8)	0.002

Contact with a person with risk factor (%)*	23 (29)	9(11.7)	0.009

Antibiotic use in last 6mo (%)	17(22.1)	13(16.9)	0.54

Contact with health worker (%)	17(22.1)	10(13)	0.2

*Intravenous drug abuse, prisoner or homeless

The prevalence of nasal colonization with MRSA was significantly higher (p ≤ 0.001) in the household contacts of the study group (18/77 or 23%) versus the control group (3/77 or 3.9%). *S. aureus *nasal colonization was detected in 6 of 24 (25%) CA-MRSA index patients; of those 5 were MRSA. The prevalence of nasal colonization with *S. aureus *was 28/77 (36%) in the household contacts of the study group versus 16/77 (21%) in the control group (p = 0.032). In the study group, all contacts from 7 families were tested negative for staphylococcal nasal colonization. The prevalence of nasal colonization with methicillin-susceptible *S. aureus *was similar between household contacts in both groups: 10/77 (13%) in the study group versus 13/77 (16.9%) in the control group. When comparing families of the two groups, 14/24 (58%) families in the study group had at least one household member nasally colonized with CA-MRSA compared to 2/29 (6.9%) in the control group (p ≤ 0.001).

Because of these differences between the household contacts of the study and control groups (Table 1), multivariate regression analysis was used to assess whether MRSA colonization was independent of these demographic variables. Non African-American ethnicity was predictive of being colonized with MRSA with OR 0.20 [95% CI 0.042-0.998], p = 0.05. The other variables including sharing towels or being in contact with a person with a risk factor were not predictive of MRSA colonization. In addition, in our study population, the following criteria were not predictive of MRSA colonization in household contacts: age, gender, history of prior SSTI, history of using public pool or parks, sharing clothes or brushes, contact with health care worker, history of use of antibiotic in the last 6 months, and owning a pet.

Twenty four clinical isolates from 24 patients of the study group were available for SCC*mec *typing and the detection of PVL genes. Three of these isolates were PVL negative and SCC*mec *II, typically associated with HA-MRSA infections. However, none of the patients presented traditional risk factors for HA-MRSA. The remaining 21 had SCC*mec *IV, typically seen in patients with CA-MRSA infections and were PVL-positive. When comparing the SCC*mec *typing within the families having at least one individual colonized with MRSA, household members shared the same SCC*mec *type as the index case in 13/14 families.

Pulse Field Gel Electrophoresis (PFGE) was used to evaluate the clonal distribution of total of 69 isolates. Based on a dice's coefficient cutoff value of 75%, we identified seven lineage clusters that could be related to the seven USA types. Of those isolates, 40 (58%) were related to USA300, nine were related to USA200, six related to USA300/700, five related to USA600, five related to USA800, two to USA100, and one of each to USA400, USA500. We were not able to relate one MSSA isolate to any of the seven lineage groups identified. Of the MRSA isolates recovered from sites of infection, 22/24 were USA300; one was USA300/700 (from patient with CSOM) and the other was not available for typing. The DNA macrorestriction profile analysis demonstrated that the SA isolates that were recovered from sites of infection were similar to those recovered from the anterior nares in 5/6 CA-MRSA patients. Among the families with at least one household contact colonized with MRSA, similar pulsovars were found between the index patient and at least one household contact in 10/14 families (USA300 in 9 families and USA300/700 in one family). Among these 14 families, USA300 was detected in 13/14 (93%) families. The frequency of USA300 isolates from family members of both groups is shown in Figure 1. The Only 1/16 SA isolates typed USA300 in the control group. Of all tested isolates, PVL genes detected in 30/52 (58%).

## Discussion

Our results demonstrated that colonization with *S. aureus *was significantly higher among MRSA patients' contacts (36%) compared to contacts of patients without *S. aureus *suspected infections (21%) (p = 0.032), but comparable to *S. aureus *nasal carriage in the US population (32.4%) reported in 2001-2002 survey [27]. The study also showed that the prevalence of MRSA colonization was significantly higher in the patients' contacts group (23%) than the control group (3%) (p < 0.001) during the same study period; and also higher than previously reported rate in the general population (0.8%) [27]. All together, these results suggest an increased prevalence of MRSA nasal colonization in the household contacts of patients with CA-MRSA infections. The prevalence of nasal colonization among all household contacts of children with CA-MRSA infections in the United States has not been previously reported. To our knowledge, the present study is the first age-matched simultaneous patient-control study done in the pediatric population.

In contrast to the CA-MRSA colonization, we found no significant difference in MSSA colonization between the two groups (p > 0.1). The source of most CA-MRSA infections in children is not always evident. Potential sources include contact sports, sharing towels, clothes or brushes, contact with the health care system or even owning a pet [28,29]. In our study, we have not found any specific risk factor that was predictive of MRSA colonization. The recent increase of invasive disease due to specific CA-MRSA strains such as the USA300 clone suggests that the risk of infection may be related to increased colonization among contacts in certain communities [30]. Nasal colonization with CA-MRSA in particular is associated with increased risk of developing invasive disease including skin and soft tissue infections [14-16]. This risk appears to be higher with CA-MRSA than MSSA [14-16].

Data on household contact colonization prevalence and transmission is scarce. Few studies demonstrated that family members can serve as reservoirs of CA-MRSA [18,19]. A study in Taiwan reported that 30 (25%) of 121 household contacts of children with CA-MRSA infections were colonized with CA-MRSA [31]. In addition, 94% of the colonization isolates were indistinguishable from the clinical isolates by pulse field gel electrophoresis (PFGE) suggesting a high association between colonization and infection. However, only 64% of the colonization isolates from the family contacts were indistinguishable from the clinical isolates by PFGE and only one third had distinct clones, suggesting that not all colonizing isolates are related to the index case. This study was limited by lack of inclusion of all household contacts in the screening process. In a study of nasal colonization with CA-MRSA among patients and their family contacts, Zafar et al. found that 41% of patients and 20% of household members were colonized with MRSA [21]. However, most of these patients were adults with SSTI. In addition, the study was limited by the small number of subjects and also by not including all household contacts in the colonization screening.

Studies have demonstrated the prevalence of different genotypes among contacts of patients with CA-MRSA [21,31]. In our study, direct spread between family contacts cannot completely explain the increased colonization of MRSA as different strain lineages were isolated among some of our study group families. Taken all together, data suggest that multiple strains of MRSA may prevail in a certain family or household. Recognition that family members may serve as reservoirs for MRSA raises important issues for infection control.

Our study displays some limitations. It was a single pediatric tertiary care center study that enrolled only hospitalized patients. In addition, the lack of screening for *S. aureus *colonization at other sites such as oropharynx, rectum and skin folds may have underestimated the true rates of colonization among patients and contacts. The follow up time between participating families was variable which might have affected the results as some individuals might be chronic carriers of MRSA while others carry it intermittently. In our study, non African-American ethnicity was predictive of being colonized with MRSA with OR of 0.20 [95% CI 0.042-0.998], p = 0.05. Because the contact (study) group included more non-African Americans, some caution should be exercised in the interpretation of the results. However, the study demonstrated the increased prevalence in MRSA nasal colonization in patients' contacts compared to age-matched controls. Although the conclusions based on this data were statistically significant, a larger set of data will strengthen these findings.

Studying the prevalence of CA-MRSA among family members of patients will help implement new prevention strategies. Our study revealed that 58% of the families of patients with CA-MRSA infections had at least one family member nasally colonized with CA-MRSA, compared to 4% in the control group (p ≤ 0.001). Thus, advice about personal/hand hygiene and environmental decontamination should be emphasized. In addition, consideration may be given to implement decolonization with topical mupirocin ointment to the anterior nares. Because of the concerns about development of resistance with increase in mupirocin use, screening of family contacts and selective decolonization may be a reasonable approach. Whether such measure will be effective for infants and young children is not clear. A study of MRSA carriage in children at a day care center suggested that throat and perianal colonization was higher than that of the nose [10]. Thus, further studies including larger number of patients and screening of colonization at different sites including the rectum, pharynx and axilla will be needed to identify how effective different decolonization measures will be. Whether nasal decolonization alone, decolonization using systemic antibiotics or a combination of both will be most effective is yet to be determined.

## Conclusion

This study showed that nasal carriage of MRSA was present in one-fourth of household contacts of children with CA-MRSA infections. Additionally, among families who had at least one household contact positive for CA-MRSA, 71.4% of those families had individuals colonized by isolates belonging to the same cluster as determined by PFGE, suggesting interfamilial transmission. This high rate of colonization and transmission may provide a rationale for more studies to investigate whether decolonization of the family contacts would be an effective control measure in preventing recurrent SSTI.

## Competing interests

The authors declare that they have no competing interests.

## Authors' contributions

YR, NAH: conception and design; acquisition, analysis and interpretation of data; drafting the manuscript. BA: conception and design; interpretation of data; drafting the manuscript. TS: patient recruitment, collection of samples, interpretation and analysis of data. CV, MJR: PFGE analysis, interpretation of data, drafting of the manuscript. MA: acquisition, analysis and interpretation of data; drafting the manuscript. All authors read and approved the final manuscript.

## Pre-publication history

The pre-publication history for this paper can be accessed here:

http://www.biomedcentral.com/1471-2334/12/45/prepub

## References

[B1] Adcock PM Pastor P Medley F Patterson JE Murphy TV Methicillin-resistant Staphylococcus aureus in two child care centers J Infect Dis 1998 178 2 577 580 9697748 10.1086/517478

[B2] Herold BC Immergluck LC Maranan MC Lauderdale DS Gaskin RE Boyle-Vavra S Leitch CD Daum RS Community-acquired methicillin-resistant Staphylococcus aureus in children with no identified predisposing risk Jama 1998 279 8 593 598 10.1001/jama.279.8.593 9486753

[B3] Lindenmayer JM Schoenfeld S O'Grady R Carney JK Methicillin-resistant Staphylococcus aureus in a high school wrestling team and the surrounding community Arch Intern Med 1998 158 8 895 899 10.1001/archinte.158.8.895 9570176

[B4] Lu PL Chin LC Peng CF Chiang YH Chen TP Ma L Siu LK Risk factors and molecular analysis of community methicillin-resistant Staphylococcus aureus carriage J Clin Microbiol 2005 43 1 132 139 10.1128/JCM.43.1.132-139.2005 15634961 PMC540160

[B5] Naimi TS LeDell KH Como-Sabetti K Borchardt SM Boxrud DJ Etienne J Johnson SK Vandenesch F Fridkin S O'Boyle C Comparison of community- and health care-associated methicillin-resistant Staphylococcus aureus infection Jama 2003 290 22 2976 2984 10.1001/jama.290.22.2976 14665659

[B6] Creech CB Kernodle DS, Alsentzer A, Wilson C, Edwards KM: Increasing rates of nasal carriage of methicillin-resistant Staphylococcus aureus in healthy children Pediatr Infect Dis J 2005 24 7 617 621 10.1097/01.inf.0000168746.62226.a4 15999003

[B7] Hussain FM Boyle-Vavra S Daum RS Community-acquired methicillin-resistant Staphylococcus aureus colonization in healthy children attending an outpatient pediatric clinic Pediatr Infect Dis J 2001 20 8 763 767 10.1097/00006454-200108000-00009 11734738

[B8] Kazakova SV Hageman JC Matava M Srinivasan A Phelan L Garfinkel B Boo T McAllister S Anderson J Jensen B A clone of methicillin-resistant Staphylococcus aureus among professional football players N Engl J Med 2005 352 5 468 475 10.1056/NEJMoa042859 15689585

[B9] Seybold U Kourbatova EV Johnson JG Halvosa SJ Wang YF King MD Ray SM Blumberg HM Emergence of community-associated methicillin-resistant Staphylococcus aureus USA300 genotype as a major cause of health care-associated blood stream infections Clin Infect Dis 2006 42 5 647 656 10.1086/499815 16447110

[B10] Shahin R Johnson IL Jamieson F McGeer A Tolkin J Ford-Jones EL Methicillin-resistant Staphylococcus aureus carriage in a child care center following a case of disease. Toronto Child Care Center Study Group Arch Pediatr Adolesc Med 1999 153 8 864 868 10437762 10.1001/archpedi.153.8.864

[B11] Miller LG Quan C Shay A Mostafaie K Bharadwa K Tan N Matayoshi K Cronin J Tan J Tagudar G A prospective investigation of outcomes after hospital discharge for endemic, community-acquired methicillin-resistant and -susceptible Staphylococcus aureus skin infection Clin Infect Dis 2007 44 4 483 492 10.1086/511041 17243049

[B12] Chen AE Cantey JB Carroll KC Ross T Speser S Siberry GK Discordance between Staphylococcus aureus nasal colonization and skin infections in children Pediatr Infect Dis J 2009 28 3 244 246 10.1097/INF.0b013e31818cb0c4 19165132

[B13] Lee MC Rios AM Aten MF Mejias A Cavuoti D McCracken GH Jr Hardy RD: Management and outcome of children with skin and soft tissue abscesses caused by community-acquired methicillin-resistant Staphylococcus aureus Pediatr Infect Dis J 2004 23 2 123 127 10.1097/01.inf.0000109288.06912.21 14872177

[B14] Davis KA Stewart JJ Crouch HK Florez CE Hospenthal DR Methicillin-resistant Staphylococcus aureus (MRSA) nares colonization at hospital admission and its effect on subsequent MRSA infection Clin Infect Dis 2004 39 6 776 782 10.1086/422997 15472807

[B15] Oztoprak N Cevik MA Akinci E Korkmaz M Erbay A Eren SS Balaban N Bodur H Risk factors for ICU-acquired methicillin-resistant Staphylococcus aureus infections Am J Infect Control 2006 34 1 1 5 10.1016/j.ajic.2005.07.005 16443085

[B16] Ellis MW Hospenthal DR Dooley DP Gray PJ Murray CK Natural history of community-acquired methicillin-resistant Staphylococcus aureus colonization and infection in soldiers Clin Infect Dis 2004 39 7 971 979 10.1086/423965 15472848

[B17] Faden H Ferguson S Community-acquired methicillin-resistant Staphylococcus aureus and intrafamily spread of pustular disease Pediatr Infect Dis J 2001 20 5 554 555 10.1097/00006454-200105000-00023 11368124

[B18] Ho PL Cheung C Mak GC Tse CW Ng TK Cheung CH Que TL Lam R Lai RW Yung RW Molecular epidemiology and household transmission of community-associated methicillin-resistant Staphylococcus aureus in Hong Kong Diagn Microbiol Infect Dis 2007 57 2 145 151 10.1016/j.diagmicrobio.2006.08.003 16989976

[B19] Huijsdens XW van Santen-Verheuvel MG Spalburg E Heck ME Pluister GN Eijkelkamp BA de Neeling AJ Wannet WJ Multiple cases of familial transmission of community-acquired methicillin-resistant Staphylococcus aureus J Clin Microbiol 2006 44 8 2994 2996 10.1128/JCM.00846-06 16891525 PMC1594612

[B20] Wagenvoort JH De Brauwer EI Sijstermans ML Toenbreker HM Risk of re-introduction of methicillin-resistant Staphylococcus aureus into the hospital by intrafamilial spread from and to healthcare workers J Hosp Infect 2005 59 1 67 68 10.1016/j.jhin.2004.07.025 15571856

[B21] Zafar U Johnson LB Hanna M Riederer K Sharma M Fakih MG Thirumoorthi MC Farjo R Khatib R Prevalence of nasal colonization among patients with community-associated methicillin-resistant Staphylococcus aureus infection and their household contacts Infect Control Hosp Epidemiol 2007 28 8 966 969 10.1086/518965 17620245

[B22] Lowell GDR LONG SS PL, Prober CG Staphylococcus aureus Principles and Practice of Pediatric Infectious Diseases 2009 3 Churchill Livingstone/Elsevier 679 693

[B23] Oliveira DC de Lencastre H Multiplex PCR strategy for rapid identification of structural types and variants of the mec element in methicillin-resistant Staphylococcus aureus Antimicrob Agents Chemother 2002 46 7 2155 2161 10.1128/AAC.46.7.2155-2161.2002 12069968 PMC127318

[B24] Lina G Piemont Y Godail-Gamot F Bes M Peter MO Gauduchon V Vandenesch F Etienne J Involvement of Panton-Valentine leukocidin-producing Staphylococcus aureus in primary skin infections and pneumonia Clin Infect Dis 1999 29 5 1128 1132 10.1086/313461 10524952

[B25] McDougal LK Steward CD Killgore GE Chaitram JM McAllister SK Tenover FC Pulsed-field gel electrophoresis typing of oxacillin-resistant Staphylococcus aureus isolates from the United States: establishing a national database J Clin Microbiol 2003 41 11 5113 5120 10.1128/JCM.41.11.5113-5120.2003 14605147 PMC262524

[B26] Tenover FC Arbeit RD Goering RV Mickelsen PA Murray BE Persing DH Swaminathan B Interpreting chromosomal DNA restriction patterns produced by pulsed-field gel electrophoresis: criteria for bacterial strain typing J Clin Microbiol 1995 33 9 2233 2239 7494007 10.1128/jcm.33.9.2233-2239.1995 PMC228385

[B27] Kuehnert MJ Kruszon-Moran D Hill HA McQuillan G McAllister SK Fosheim G McDougal LK Chaitram J Jensen B Fridkin SK Prevalence of Staphylococcus aureus nasal colonization in the United States, 2001-2002 J Infect Dis 2006 193 2 172 179 10.1086/499632 16362880

[B28] Redziniak DE Diduch DR Turman K Hart J Grindstaff TL MacKnight JM Mistry DJ Methicillin-resistant Staphylococcus aureus (MRSA) in the Athlete Int J Sports Med 2009 30 8 557 562 10.1055/s-0029-1214382 19468969

[B29] Walther B Wieler LH Friedrich AW Hanssen AM Kohn B Brunnberg L Lubke-Becker A Methicillin-resistant Staphylococcus aureus (MRSA) isolated from small and exotic animals at a university hospital during routine microbiological examinations Vet Microbiol 2008 127 1-2 171 178 10.1016/j.vetmic.2007.07.018 17804179

[B30] Abdel-Haq N Al-Tatari H Chearskul P Salimnia H Asmar BI Fairfax MR Amjad M Methicillin-resistant Staphylococcus aureus (MRSA) in hospitalized children: correlation of molecular analysis with clinical presentation and antibiotic susceptibility testing (ABST) results Eur J Clin Microbiol Infect Dis 2009 28 5 547 551 10.1007/s10096-008-0658-4 19020911

[B31] Huang YC Ho CF Chen CJ Su LH Lin TY Nasal carriage of methicillin-resistant Staphylococcus aureus in household contacts of children with community-acquired diseases in Taiwan Pediatr Infect Dis J 2007 26 11 1066 1068 10.1097/INF.0b013e31813429e8 17984820

